# Laser-Induced Highly Stable Conductive Hydrogels for Robust Bioelectronics

**DOI:** 10.1007/s40820-024-01519-w

**Published:** 2024-11-05

**Authors:** Yibo Li, Hao Zhou, Huayong Yang, Kaichen Xu

**Affiliations:** State Key Laboratory of Fluid Power and Mechatronic Systems, School of Mechanical Engineering, Zhejiang University, Hangzhou, 310058 People’s Republic of China

**Keywords:** Laser processing, Conductive hydrogels, Stable interface, Bio-interfacing electrodes, Bioelectronic application

## Abstract

**Abstract:**

Despite the promising progress in conductive hydrogels made with pure conducting polymer, great challenges remain in the interface adhesion and robustness in long-term monitoring. To address these challenges, Prof. Seung Hwan Ko and Taek-Soo Kim’s team introduced a laser-induced phase separation and adhesion method for fabricating conductive hydrogels consisting of pure poly(3,4-ethylenedioxythiophene):polystyrene sulfonate on polymer substrates. The laser-induced phase separation and adhesion treated conducting polymers can be selectively transformed into conductive hydrogels that exhibit wet conductivities of 101.4 S cm^−1^ with a spatial resolution down to 5 μm. Moreover, they maintain impedance and charge-storage capacity even after 1 h of sonication. The micropatterned electrode arrays demonstrate their potential in long-term in vivo signal recordings, highlighting their promising role in the field of bioelectronics.

**Graphical Abstract:**

## Highlights

- Stable adhesion of pure poly(3,4-ethylenedioxythiophene):polystyrene sulfonate hydrogel to polymer substrates was successfully achieved via a laser-induced phase separation and adhesion method.
- The resulting conductive hydrogel exhibits a superior wet electrical conductivity up to 101.4 S cm^−1^ and a spatial resolution down to 5 μm.
- Such hydrogels hold great promise in robust bio-interfacing electrodes suitable for long-term high-fidelity signal monitoring.

## Graphical abstract

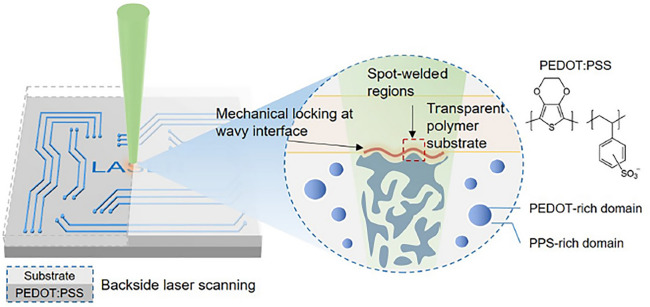

## Introduction

Advances in soft materials have significantly boosted the development of bio-interfacing electrodes, attracting widespread attention in the fields of implantable bioelectronics, wearable medical devices, and brain-machine interfaces. Conductive hydrogels stand out for their mechanical properties that are closer to biological tissues [1]. Particularly, conductive hydrogels using pure conducting polymers poly(3,4-ethylenedioxythiophene):polystyrene sulfonate (PEDOT:PSS) emerge as a promising candidate among soft interfacing materials due to their excellent biocompatibility, electrochemical performance and mechanical properties [2]. However, the stability and interfacial adhesion remain a challenge in wet physiological environment, which may lead to delamination issues. In addition, high-resolution patterning techniques are also required to achieve high-fidelity recording sites with a high density of arrays for long-term real-time monitoring. To address these issues, Won et al. proposed an innovative approach to fabricating bio-interfacing electrodes of PEDOT:PSS hydrogels on polymer substrates that offer exceptional wet adhesion via a laser-induced process [3].

**Fig. 1:**
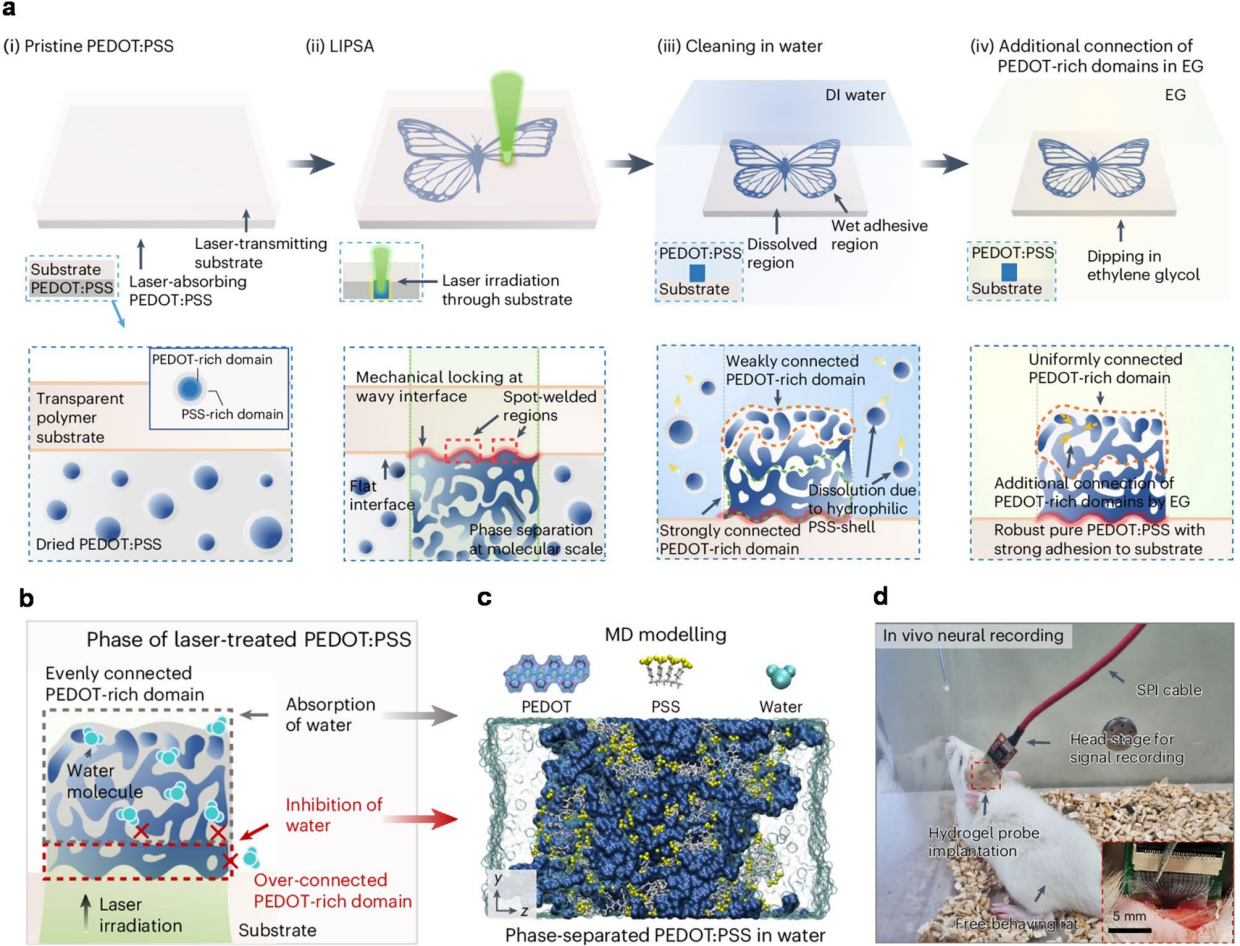
Laser-induced PEDOT:PSS hydrogels with strong wet stability and adhesion for robust bioelectronics. **a** Schematic of the LIPSA method for fabricating conductive PEDOT:PSS hydrogel on polymer substrates. **b** Schematic of the degree of phase separation in the thickness direction. **c** Framework of MD simulations. **d** In vivo neural recording scenario of a free-behaving rat. Inset: Implantation region for hydrogel probes. Adapted from Ref. [3]

Laser processing technique, known for its unique characteristics of the strong electric field and photothermal energy, holds great promise in material modification and high-resolution patterning [4]. To fully leverage the potential of laser, Seung Hwan Ko and the collaborators introduced the laser-induced phase separation and adhesion (LIPSA) method for fabricating conductive hydrogels on polymer substrates (Fig. 1a). This technique employs the backside laser scanning of the interface between PEDOT:PSS and the polymer substrate, leveraging the local thermal effect to induce phase separation. The process is further refined by post-treatment with ethylene glycol, which optimizes the dispersion of PEDOT within the hydrogel matrix. The combined processes not only enhance the connection between PEDOT-enriched areas but also induce mechanical locking structures and spot-welded regions within the wavy interface, which significantly improves the wet stability, adhesion and simultaneously provides high conductivity for the hydrogels. The LIPSA method results in conductive hydrogels with a superior wet conductivity (101.4 S cm^−1^ in wet state) and a spatial resolution down to 5 μm, rendering them suitable for reusable, soft bio-interfacing electrodes. Moreover, the method achieves remarkable peel strength of 64.4 N m^−1^ and lap-shear strength of 62.1 kPa, demonstrating the robust adhesion of the hydrogels to the polymer substrates.

The enhanced wet stability and adhesion of the PEDOT:PSS hydrogel in wet conditions are attributed to sophisticated interactions between the material composition and microstructure. On the one hand, the PEDOT:PSS hydrogel consists of two regions in the thickness direction where the formation of a hydrophobic PEDOT-rich domain serves as a wet adhesion promoter, hindering water penetration at the interface (Fig. 1b). The hydrophobic nature of this domain is a key factor in the hydrogel's wet adhesion, as it reduces the interfacial delamination when exposed to physiological conditions. Phase images captured by atomic force microscopy confirm the aforementioned theory that the strongly connected PEDOTs are separated from the PSS to form large grains in the water-inhibiting region. On the other hand, molecular dynamic (MD) simulations reveal a unique interaction between the PEDOT and PSS components at a molecular level. The PEDOT:PSS system exhibits a morphological feature where the sulfonated groups of PSS are found to engage in strong interactions with adjacent PEDOT chains (Fig. 1c), thus hindering the diffusion of water into the interior regions and preventing the swelling and degradation that would otherwise be induced by moisture. This dual mechanism is a testament to the innovative design of the hydrogel, which addresses the longstanding challenge of creating bio-interfacing electronics with both high durability and electrical performance. The researchers quantitatively assessed the LIPSA technique's robust adhesion in wet conditions through 90° peel-off and lap-shear tests. The wet adhesion improved with the increase of laser power in LIPSA, culminating in an average peak of 61.5 N m^−1^ and 56.0 kPa at the optimal laser power of 250 mW. In a dry environment, the PEDOT:PSS hydrogel achieved its maximum adhesion strength of over 227 kPa when subjected to the LIPSA process at the aforementioned optimal energy threshold.

For bioelectronic applications, the researchers validated the concept of hydrogel microelectrode arrays in a rat model, demonstrating their potential for long-term in vivo signal recording in brain and heart. First, soft neural probes with 16 PEDOT:PSS hydrogel microelectrodes on polyethylene terephthalate substrate were delicately implanted into a somatosensory cortex region of the rat’s brain (Fig. 1d). Action potential of various states of the rat was precisely recorded by utilizing a high-pass filtering that ranges from 300 to 7.5 kHz (60 Hz of notch filter). This implanted neural probe reveals a stable and even increase in signal-to-noise ratio (SNR) for each data stream over 3 weeks. The enhanced SNRs are attributed to the electrode-brain tissue interface facilitated by the healing process. Additionally, strain-insensitive, highly stretchable, hydrogel microelectrode arrays on thermoplastic polyurethan were designed in the same procedure to record epicardial cardiac signals. The filtering frequency was adjusted to 1–200 Hz (60 Hz of notch filter). It is worth noting that these hydrogel microelectrodes can even undergo ultrasonic cleaning (1 h) with almost no reduction in signal quality after cleaning. It is expected for development of reusable electronic devices. These two scenarios demonstrate the potential for microelectrode patterning on different elastomers to achieve long-term robust monitoring.

Although the proposed LIPSA technique presents great potentials, additional efforts should be involved for practical and universal applications. On this basis, profound perspectives for enhancing the PEDOT:PSS based bio-interfacing electrodes were put forward in detail: (i) high-density electrodes array patterning: High spatiotemporal resolution of neural probe plays a crucial role in neural recordings such as electrocorticography, as the spatial scale of some pathologic signals is at the submillimeter or micrometer level; (ii) electrical conductivity enhancement of conductive hydrogels: Applying this bio-interfacing electrode to implantable bioelectronics devices requires further research into the ability to capture high-fidelity biosignals. This requires further enhancement of its electrical conductivity and robustness during long-term monitoring; (iii) electrodes arrays based on soft actuators: In the future, the integration of LIPSA-treated hydrogels with soft actuators could be a groundbreaking development, providing the electrodes with ability to conform to complex and varied biological structures. The incorporation of such on-demand deformation capabilities would not only enhance their adaptability and comfort in complex physiological environments but also allow for self-healing capabilities, thereby extending their service life and reliability in dynamic and even harsh physiological environments.

In conclusion, by leveraging laser-induced phase separation, PEDOT:PSS hydrogels can stably and selectively adhere to various polymer substrates in wet environments and have demonstrated capabilities in long-term in vivo monitoring of neural electrical signals. Such LIPSA method offers pivotal avenues for the development of soft bio-interfacing electrodes which are reusable, robust and interfacial stable in wearable and implantable bioelectronics.

## References

[1] Y. Lu, G. Yang, S. Wang, Y. Zhang, Y. Jian et al., Stretchable graphene–hydrogel interfaces for wearable and implantable bioelectronics. Nat. Electron. **7**, 51–65 (2023). 10.1038/s41928-023-01091-y

[2] D. Won, J. Kim, J. Choi, H.J. Kim, S. Han et al., Digital selective transformation and patterning of highly conductive hydrogel bioelectronics by laser-induced phase separation. Sci. Adv. **8**, eabo3209 (2022). 10.1126/sciadv.abo3209 35675404 10.1126/sciadv.abo3209 PMC9177068

[3] D. Won, H. Kim, J. Kim, H. Kim, M.W. Kim et al., Laser-induced wet stability and adhesion of pure conducting polymer hydrogels. Nat. Electron. **7**, 475–486 (2024). 10.1038/s41928-024-01161-9

[4] Y. Kim, E. Hwang, C. Kai, K. Xu, H. Pan et al., Recent developments in selective laser processes for wearable devices. Bio-Des. Manuf. **7**, 517–547 (2024). 10.1007/s42242-024-00300-7

